# Could COVID-19′s Aftermath on Children’s Health Be Felt into the 22nd Century?

**DOI:** 10.3390/children9040482

**Published:** 2022-04-01

**Authors:** Alexios-Fotios A. Mentis, George Paltoglou, Panayotes Demakakos, Faheem Ahmed, George P. Chrousos

**Affiliations:** 1 University Research Institute of Maternal and Child Health and Precision Medicine, National and Kapodistrian University of Athens, 11527 Athens, Greece; gpaltoglou@gmail.com (G.P.); chrousos@gmail.com (G.P.C.); 2 Adolescent Health Care, National and Kapodistrian University of Athens, “Aghia Sophia” Children’s Hospital, 11527 Athens, Greece; 3 Division of Endocrinology, Metabolism and Diabetes, First Department of Paediatrics, National and Kapodistrian University of Athens Medical School, “Aghia Sophia” Children’s Hospital, 11527 Athens, Greece; 4 Department of Epidemiology and Public Health, University College London, London WC1E 6BT, UK; p.demakakos@ucl.ac.uk; 5 NHS England, London SE1 6LH, UK; faheem.ahmed@doctors.org.uk; 6 Graduate School of Business, Columbia University, New York, NY 10027, USA

**Keywords:** COVID-19, developmental origins of disease, obesity, stress, children’s health, malnutrition, adolescence, pandemics, inequity

## Abstract

The COVID-19 pandemic has massively affected people’s health, societies, and the global economy. Our lives are no longer as they were before COVID-19, and, most likely, will never be the same again. We hypothesize that the effect of the COVID-19 pandemic on population health and the economy will last for a very long time and will still be felt in the 22nd century. Our hypothesis is based on evidence from the 1918–1919 influenza pandemic, the Dutch famine during the Second World War, and the 2007–2008 economic crisis, as well as from the rationally predicted impact of COVID-19 on human development. We expect that the COVID-19 pandemic, including the mitigation measures taken against it, will affect children’s development in multiple ways, including obesity, both while in utero and during critical and sensitive windows of development, including the early childhood years and those of puberty and adolescence. The psychosocial and biological impact of this effect will be considerable and unequally distributed. The implications will last at least a lifetime, and, through inter-generational transmission, will likely take us to future generations, into the 22nd century. We argue for the urgent need of designing and initiating comprehensive longitudinal cohort studies to closely monitor the long-term effects of COVID-19 on children conceived, born, and raised during the pandemic. Such an approach requires a close and effective collaboration between scientists, healthcare providers, policymakers, and the younger generations, and it will hopefully uncover evidence necessary to understand and mitigate the impact of the pandemic on people’s lives in the 21st and 22nd centuries.

## Main Manuscript

The COVID-19 pandemic has had unprecedented human, societal, and financial consequences, ranging from loss of life (at the level of tens of millions excess years of life in several countries), to severe mental health issues [1], as well as marked increases in poverty and inequity [2] and major disruptions of global markets [3,4,5]. The societal and psychological effects of the COVID-19 pandemic is particularly felt by vulnerable populations, such as children (even though this age group is the least affected one by direct, health-related SARS-CoV-2 effects) and those suffering from mental health disorders or other chronic conditions [6,7,8]. Irrespective of when the COVID-19 pandemic will end [9], it is likely that its impact on children’s development will persist for many decades. Importantly, the decades-long COVID-19 effects will not merely refer to long COVID (i.e., post-acute or long-lasting medical sequelae), or to whether the SARS-CoV-2 virus will become endemic, or even to the collective memories that will capture this and future generations [10]. Rather, it holds true especially amidst the hovering uncertainty regarding economic sustainability during the 21st century [11] and beyond.

Here, we move one step forward to hypothesize that, regardless of the evolution and progression of the pandemic, the disease will quite likely continue to have negative health and societal influences throughout the 21st and well into the 22nd century. These consequences will be related to the biological effects on the physical and mental stress in pregnant women, as well as infants, children, and adolescents born and/or raised and educated during the COVID-19 pandemic. On the other hand, they will be pertinent to the inequity in psychosocial development and in the distribution of access to education, both of which will be exacerbated at least by the COVID-19 pandemic, potentially in a syndemic manner [8] (as in other settings [12]). Our hypothesis is based on evidence of long-term consequences arising out of earlier pandemics (like the 1918–1919 influenza pandemic) and other major crises (such as the Dutch famine in the Second World war, and the 2007–2008 economic crisis), whose effects persist even today [13]. Indispensable to testing this hypothesis will be the COVID-19 birth and children’s cohort studies that will hopefully be conducted at a multinational level, as we discuss below.

There are plenty of data to support our hypothesis. The in utero exposure to influenza during the 1918–1919 pandemic profoundly impacted the population of the United States after 1940 [14]; the population exposed to influenza in utero showed significantly lower participation in educational activities (15% lower rates of high-school graduation), physical status (~20% higher rates of disability), socio-economic status (~15% increase in risk of being trapped in poverty), and income (5–9% lower wages in men), and higher levels of type 2 diabetes mellitus, heart disease, and overall mortality in the following decades [14,15]. The Dutch famine led to inter-generational increases in obesity and metabolic syndrome manifestations [16]. The 2007–2009 financial crisis led to excess mortality (increased mortality per year since then) and other adverse outcomes, ranging from nutritional deficiencies in children, major food insecurity, suicides and mental health disorders [17,18]. We contend that the post-pandemic period will be referred to as the double E: an epidemiological and economic crisis.

We expect that the pandemic itself and the mitigation measures taken will have long-term health consequences by severely affecting individuals early in life, starting with their antenatal lives as can be derived from the theory of the in utero origins of adult disease [19]. Accordingly, markers of in utero development, such as maternal stress and intrauterine growth, length of gestation, birth weight, and so on, can be causally linked to several diseases in adulthood (e.g., obesity, metabolic syndrome, hypertension, cardiovascular diseases, accelerated aging and cancer), with relevant observations in both humans and rodents [20,21,22,23]. Epidemiologists and economists have also long recognized the critical influences that fetal, infancy, and early childhood environments have on diseases of adulthood, as well as on how attenuated cognitive skills due to harsh childhood settings can lead to reduced salaries and wages [24]. Therefore, major protracted events, such as infectious pandemics or financial crises, can have a long-term impact if and when they affect in utero and childhood development. Of note, the human organism is in development until the middle of the third decade of life, when the brain white matter, i.e., neuronal connectivity, is fully completed.

Could these and similar outcomes and theories be applicable to the ongoing COVID-19 pandemic? We believe yes. To begin with, even correcting for genetic and environmental factors, psychosocial stress in pregnant women increases the risk of maternal mental health problems and may have life-long adverse health consequences in their offspring (for a review, see [25]). It is likely, on the one hand, that the number of preterm births during the pandemic is paradoxically reduced, perhaps because pregnant women safely are home-bound for long periods, thereby, reducing harmful exposures, while, on the other hand, parents stay at home longer than usual and, thus, interact with their children more. Nonetheless, it is questionable if these effects would compensate for the pandemic-related stress over the long term [15].

The colossal financial consequences of the COVID-19 pandemic are likely to further widen socioeconomic inequities and increase poverty, in turn worsening the vicious cycle of inter-generational poverty and its adverse impact on human health. A reduction in educational attainment during the pandemic will exacerbate underlying determinants of poor health, such as low socioeconomic status, especially in disadvantaged and minority ethnic groups [26]. Importantly, the need for accelerated digitalisation, with tele-teaching in school education and tele-care in health care systems, may increase rather than diminish the socioeconomic inequity, as observed during the COVID-19 pandemic [27]. Collectively, it is highly possible that the profound biological, psychological, economic, and societal impact of COVID-19 on childhood will create persistent health and societal problems for many decades to come, extending even into the 22nd century.

There is solid biological evidence to support the above claims. Notably, the adverse impacts on fetal development could be mediated by several, well-established psycho-neuro-endocrinological associations linking the brain with the endocrine system and the peripheral tissues; for instance, the hyper- or hypo-secretion of corticotropin-releasing hormone acting via the pituitary-adrenal axis, the prolonged activation of the locus caeruleus-norepinephrine system acting via the sympathetic and parasympathetic nervous systems, the remodeling of brain structures (e.g., the amygdala and hippocampus), the persistent neuroendocrine dysregulation, and multiple epigenetic alterations [25,28]. Likewise, inter-generationally transmitted stress (e.g., caused by poverty or violence-related trauma) may also have a biological basis, including dysregulated glucocorticoid signalling, epigenetic changes in DNA and chromatin and alterations in small or long non-protein coding RNA molecules [29,30].

This prompts a logical and major public health-oriented question: should we adopt a wait-and-see approach as a form of management of the long-term effects of the pandemic, or do something actively? This question is especially relevant to the life expectancy of those conceived, born and raised during the pandemic, which had been estimated to be >95 years in many developed countries prior to the pandemic [31]. Following analogous approaches for today’s adolescents [32] and adults [33], we contend that longitudinal cohort studies are essential to closely monitor the impact of the COVID-19 pandemic on children conceived, born and raised during the pandemic, as it regards their future development into adulthood and their adult functioning.

How do we achieve this? While providing early proof (or at least evidence) for supporting this hypothesis on the lasting effects into the 22nd century is difficult, we and others argue that a multinational birth cohort study, broad in scope and comprehensive, is indisputably and urgently required [15]. There are some initial data supporting our hypothesis. For instance, the rates of increase in children’s and adolescents’ body mass index and type 2 diabetes mellitus have practically doubled during vs. before the COVID-19 pandemic, and would be hardly restored even if lockdown-related sedentarism is attenuated [33,34].

The suggested multidisciplinary birth and children’s cohort study should ideally include a comparison of current neonates, infants, and adolescents across continents experiencing variable infectivity rates and those born in the few years before the COVID-19 pandemic. Its main objective should be to explore whether the COVID-19 pandemic has exerted long-term effects on human health and well-being. Such studies should provide a meticulous description and assessment of potential causal, confounding and mediating factors, they should focus on sex-specific risk, considering that men have increased levels of mortality compared to women during pandemics and famines [35], and they should require a follow-up for at least half a century (i.e., until the subjects reach mature adulthood). Appropriate measurements must include easily measurable indices to allow for data collection in resource-poor settings that capture metrics of child development, cognitive function, educational attainment, socioeconomic position, and disease status. Considering that the in utero and childhood effects of an environmental exposure, such as the SARS-CoV-2 pandemic, may involve mechanisms other than those related to classic neurodevelopment (discussed in [36]), including trans-generational epigenetics and/or immune-mediated viral effects, continent-wide biobanks would ideally be established to collect samples to assess relevant biomarkers (e.g., CpG methylation or histone modification status, B- and T-cell receptor sequencing, etc.). In conducting such a study, ethical issues should also be considered and continually reviewed, especially if the findings mandate early intervention to mitigate against any adverse sequelae discovered in interim analyses.

While describing and analysing the above-mentioned phenomena is interesting from the epidemiological perspective, this approach is insufficient on its own to guarantee humanity’s future prosperity without full commitment by policymakers to heavily invest in the public health and future well-being of children born and growing up during the pandemic [37,38]. Given that we are already two years into the pandemic, action is required now to learn from this situation, so that we can be ready for future ones. Collaboration and understanding between policymakers, healthcare providers, epidemiologists, and data scientists are crucial. Inclusion of the younger generation of citizens as stakeholders in this agenda is imperative as well [39]. The pandemic represents an unparalleled opportunity to rethink, redesign, restructure, and re-implement our public, global and even planetary health policies and to interrupt to the best possible extent its inter-generational transmission into the future.

## Data Availability

None applicable.
